# Effects of the differences in mental states on the mirror system activities when observing hand actions

**DOI:** 10.1186/s40101-018-0192-8

**Published:** 2019-01-03

**Authors:** Yuki Ikeda, Yuki Nishimura, Shigekazu Higuchi

**Affiliations:** 1 Graduate School of Integrated Frontier Sciences, Kyushu University, 4-9-1 Shiobaru, Minami-ku, Fukuoka City, Fukuoka 8158540 Japan; 2 Japan Society for the Promotion of Science, Kojimachi Business Center Building, 5-3-1 Kojimachi, Chiyoda-ku, Tokyo, Japan; 3 Department of Human Science, Faculty of Design, Kyushu University, 4-9-1 Shiobaru, Minami-ku, Fukuoka City, Fukuoka Japan

**Keywords:** Mental states, Mirror system, Mu suppression

## Abstract

**Background:**

It is known that the activities of the mirror system are related to imitation and understanding of the intention of an action. It has been reported that the activity of the mirror system is higher for observations for imitating and understanding the intention of an action than for simple observations. However, observations that facilitate the mirror system’s activities, if they are observations intending to imitate an action or observations for understanding the intention of an action, have not been clarified to date.

**Methods:**

The types of observations of actions that highly facilitate mirror system activities were investigated. Participants were right-handed university students (*N* = 23). They observed videos showing hand actions following three types of instructions: (1) to observe the videos intending to understand the intention of the action (action understanding, AU), to observe the videos intending to imitate the hand action (imaginarily imitation, II), and to observe the videos without any intention (observation, OB). Brain waves during observation were measured, and the suppression rate of 8–10 Hz (lower mu/α) and 10–12 Hz (upper mu/α) in the central and occipital regions of the brain was calculated. The rate of suppression was compared among the conditions using a repeated measures analysis of variance for each region.

**Results:**

There was a main effect of the condition in the central region in 10–12 Hz. The degree of suppression in the AU condition was significantly larger than SO condition (*p* < 0.05) and II condition (*p* < 0.1). However, there were no differences among conditions in 8–10 Hz, the occipital region, or in either frequency band.

**Conclusions:**

These results suggest that activities of the mirror system are enhanced when observing an action with the purpose of understanding the intention of the action. Differences in the mirror system activities according to the changes of inner states might be better reflected in high-frequency mu waves.

## Background

A mirror neuron is a neuron that fires when both performing and observing an action, which were discovered in the area F5 of a macaque monkey [1]. The discovery of this neuron suggested that the processes of performing and perceiving an action might be shared in the brain. Studies using the transcranial magnetic stimulation [2], brain-imaging studies [3, 4], and studies using electroencephalogram (EEG) [5] have indicated that activation of the motor-related areas also occurs in human beings when observing another person’s action. The nervous system related to this phenomenon is called the mirror neuron system or mirror system. Brain regions such as the inferior frontal gyrus, inferior parietal lobule, and superior temporal sulcus are known to compose the mirror system [6]. The mirror system has been identified as the neural basis supporting imitation and understanding of other people’s intentions, which is a basic function required by a society [7].

Among brain wave components, the rhythms between 8 and 13 Hz that occur around the central sulcus are called mu waves. The mu wave is suppressed not only when performing an action but also when observing another person’s action. Therefore, it is used as an index of mirror system activity [8–10]. Previous studies examining correlations between functional magnetic resonance imaging and brain waves have indicated correlations between the increase in the activity rate of the inferior parietal lobule, superior parietal lobule, and the dorsal premotor cortex and the decreased rate of activity in the α-band power in the central region compared between the time of observing an action and that of performing an action [11, 12]. The brain regions showing the correlations corresponded to the areas composing mirror system, suggesting that mu waves might reflect mirror system activities. Fox et al. discussed the validity of a mu wave as an index of the mirror system through meta-analysis and concluded that a mu wave is regarded as an index with a certain level of reliability [13]. On the other hand, it has been indicated that mu waves might be affected by the α waves appearing in the occipital region, and careful examination is required [12]. Moreover, it has been suggested that mu waves reflect different functions between the lower band (8–10 Hz) and the upper band (10–12 Hz). Pfurtscheller et al. indicated that mu suppression widely occurred on the somatic sensory cortex in the lower band, regardless of the type of movement, whereas in the upper band, the suppression occurred more limitedly, depending on the type of arm movement [14]. Furthermore, it has been reported that the mu wave upper band shows specific responses, correlated with object-oriented actions [15], social interactions [16], and actions with high target orientation using hands or tools [17].

The mirror system is said to be involved in imitation of an action and understanding of the intention of an action. Caspers et al. conducted a meta-analysis of the brain regions that were activated when observing an action aiming to imitate it and when observing it without any intention, indicating many of the activated brain regions were consistent [18]. This study also suggested that mirror system activities were higher when observing with an intention of imitation. Moreover, Iacoboni et al. indicated the mirror system was activated in both simple observation and observation aiming to understand the intention of an action [19]. Furthermore, Perry et al. reported that mu suppression increased when understanding a social context based on an action, compared to when judging gender [20]. As described above, the level of mirror system activation when observing an action is considered to change depending on the inner state, i.e., whether intending to imitate the action or intending to understand the intention of the action. Previous studies compared the changes of mirror system activities with the control condition, i.e., in observation intending to imitate an action and in simple observation, as well as in observation intending to understand the intention of an action and in performing the action. It has not been clarified yet which inner state might more facilitate mirror system activities.

This study examined what type of action observation might most facilitate mirror system activities, through requesting participants to observe hand actions aiming to imitate the actions, to observe aiming to understand the action intention, and to observe without any intention.

## Methods

### Participants

Right-handed university students (*N* = 23, 15 males, and 8 females, mean age = 23.1 ± 1.0) participated in the experiment. The dominant hand was confirmed using the Edinburgh Handedness Inventory. Moreover, personality traits were examined using the Japanese version of the Interpersonal Reactivity Index [21], and there were no participants having extreme personality traits. Explanations about the experiment were provided in advance, and written consent for participating in the experiment was obtained. This experiment was conducted after obtaining approval from the ethics committee of the Graduate School of Design, Kyushu University, following the Declaration of Helsinki.

### Experimental conditions and procedures

Three conditions were prepared when observing the videos: (1) to actively observe the videos thinking about the intention of the action (action understanding, AU), (2) to actively observe the videos intending to imitate the action (imaginarily imitation, II), and (3) to passively observe the videos without any intention (simple observation, SO). The following instructions were given to the participants before showing the videos. AU condition: “Please watch the videos with thinking about in what context the action is performed.” II condition: “Please watch the videos intending to imitate the action, without moving your hands.” SO condition: “Please just observe the action.” In AU condition, to make participants actively observe the video, they were encouraged to remember inferred intentions of action as many as possible. This was ensured by telling them beforehand that we will ask about the intentions which they came up with at the end of the condition.

**Fig. 1:**
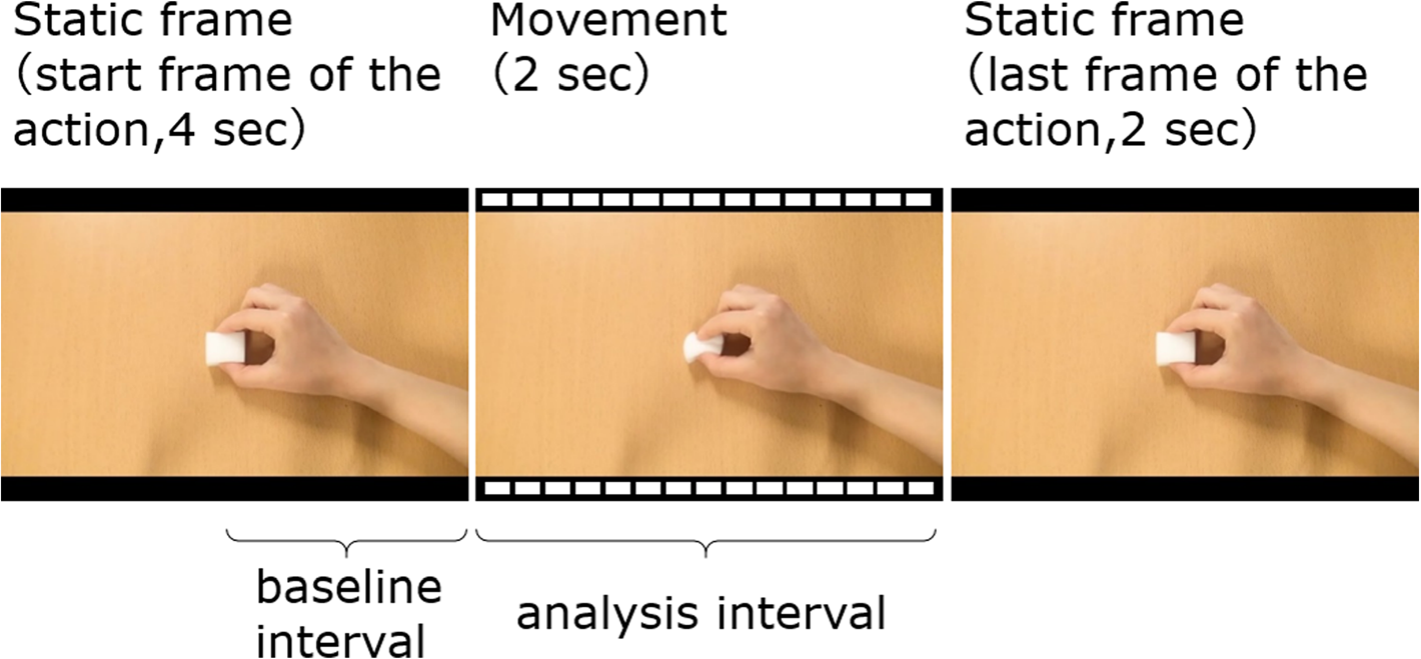
An example of the video and the ways of presentation

The observation was conducted three times. Prior to the observation, one of the three instructions was given to the participants. The video consisted of a static image for 4 s (start frame), a moving image for 2 s, and a static image for 2 s (last frame), referring to the presentation method suggested by Hobson and Bishop [22] (Fig. 1). The video was edited using Adobe Premiere Pro CC 2018 and repeatedly shown 30 times. Three types of actions using the right hand and a sponge were presented: picking up a white sponge with the index finger and middle finger, tapping the upper side of the sponge with the index finger, and rubbing the desk with the sponge. The combination of the video and instruction was randomly set depending on the participant.

Experimental procedures were as follows: after obtaining the response to IRI, electrodes for electroencephalogram (EEG) were attached to the participants’ scalp, and video observation was conducted under three types of instructions. After finishing observation under each condition, participants answered a questionnaire. Question items for subjective assessment were as follows: (1) “To what extent could you follow the instruction?” (2) “To what extent could you concentrate on the task?” (3) “How much did you feel sleepy?” and (4) “Have you ever experienced the action you observed?” Regarding (1), (2), and (3), the visual analog scale (VAS) was used for the response. Under the AU condition, participants freely described (5) “What did you think was the intention of the action?”

### EEG measurement and analysis

Brain waves were measured using 64ch EEG (Net Amps 200, EGI), with the setting of sampling 500 Hz and high-pass filter 0.01 Hz. Cz was used as a reference electrode. Following the operation manual, impedance was maintained under 50 kΩ. Stimuli were presented using Presentation Ver. 20.0 (NBS Inc.) and a 23-in. display (LG Electronics).

MATLAB 2017b (Mathworks, Inc.) and EEGLAB v14.1.2 [23] were used for analysis. As pretreatment, data obtained through measurement was filtered using a band-pass filter. Bad channels were excluded using clean_rawdata plugin of EEGLAB, which is a plug-in software of EEGLAB, and data were complemented using other channels. The reference type was changed to a common average. Epochs including values exceeding ± 500 μV and epochs including values exceeding 6 SD at single channel as well as including values exceeding 2 SD at all the channels were excluded. Data were analyzed using independent component analysis. Among the obtained components, electrooculogram, electrocardiogram, and components that obviously seem artifacts were excluded by visual assessment.

**Fig. 2:**
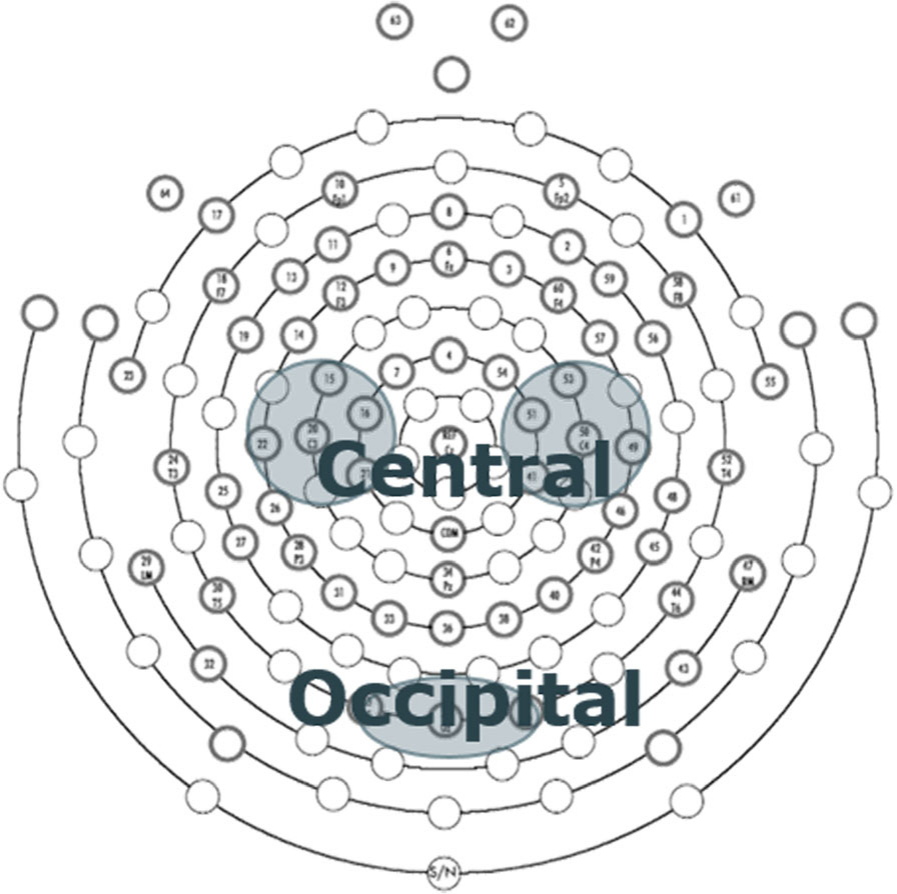
Layout of electrodes. 15, 16, 20, 21, and 22 were used in the left central region; 41, 49, 50, 51, and 53 were used in the right central region; and 35, 37, and 39 were used in the occipital region

After completing the process above, event-related spectral perturbation (ERSP) was calculated with regard to the 2-s static image before starting the moving image as the baseline. The frequency band of mu waves, which are the analysis subjects, was divided into 8–10 Hz and 10–12 Hz, referring to Pfurtscheller et al. [14]. Based on ERSP, mean values were calculated in 8–10 Hz and 10–12 Hz respectively. Moreover, the mean value during 2 s of presenting the moving image was calculated. Here, two participants that showed outliers were excluded. The regions of interest were the right and left of the central region where mu waves were observed and the occipital region where α waves were observed. The mean values of the ERSP of plural electrodes were calculated (Fig. 2). The ERSP values obtained in the right and left of the central region were regarded as mu suppression, and those obtained in the occipital region were regarded as α suppression.

### Statistics

The results of the questionnaires were analyzed using one-way analysis of variance, with regard to the condition as a factor depending on the question item, and comparison was made among three conditions. Mu suppression and α suppression were examined using a one-sample *t* test, regarding whether suppression at action observation significantly occurred compared to the baseline in the right/left side of the central region and in the occipital region respectively. Next, the amount of suppression was compared among three conditions using analysis of variance, in each region of interest. Since the stimuli were actions performed by the right hand, the central region was analyzed using two-way repeated measures analysis of variance, with the position (right/left) and the condition as factors. Regarding the occipital region, one-way repeated measures analysis of variance was conducted with the condition as a factor. For adjusting multiple comparisons, which is a sub-effect test, the Bonferroni correction was used.

## Results

### Subjective assessment using VAS

No differences were shown among three conditions in (1) engagement in the task, (2) attention to the task, and (3) sleepiness (Table 1). One participant that answered he/she had no experience of an action (4. experience of an action) and one participant that reported excessive sleepiness (3. sleepiness) were excluded from the analysis subjects.

**Table 1:** Subjective assessment using VAS (mm, mean (SD), *n* = 19)

	AU	II	SO	*F* ratio, *p* value
1. Engagement	77.9 (16.1)	75.8 (14.2)	71.6 (16.8)	*F*(2,36) = 1.03, *p* = 0.37
2. Attention	77.4 (16.3)	73.8 (17.5)	72.5 (16.0)	*F*(2,36) = 1.01, *p* = 0.38
3. Sleepness	32.7 (24.8)	37.2 (26.3)	35.5 (26.2)	*F*(2,36) = 0.92, *p* = 0.41

### mu suppression when observing the videos (the central region)

**Fig. 3:**
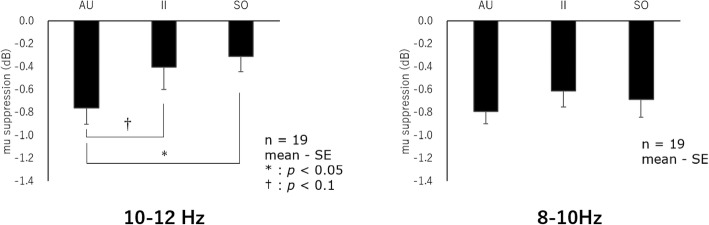
Mu suppression in the central region when observing the videos

The results of a one-sample *t* test in the central region indicated significant mu (10–12 Hz) suppression compared to the baseline in the left side under AU and SO conditions (AU: *p* < 0.001; SO: *p* = 0.012). Under II condition, suppression tended to be significant (*p* = 0.053). In the right side, mu suppression compared to the baseline was also significant under AU and SO conditions (AU: *p* < 0.001; SO: *p* = 0.031). Under II condition, suppression tended to be significant (*p* = 0.069). Next, the results of two-way analysis of variance (conditions and right/left positions as factors) indicated a significant main effect of the condition (*F*(2,36) = 3.67, *p* = 0.035). Main effects of the right/left positions as well as interactions between the right/left positions and conditions were not indicated. Therefore, a sub-effect test was conducted using the mean values of the right/left position, indicating that the suppression amount under AU condition was larger than that under SO condition (*t*(18) = 3.09, *p* = 0.019). Moreover, the suppression amount under AU condition tended to be larger than that under II condition (*t*(18) = 1.91, *p* = 0.072) (Fig. 3, left).

The results of a one-sample *t* test in the central region indicated significant mu (8–10 Hz) suppression in the left side under all the conditions (*p* < 0.001). Significant suppression was also indicated in the right side under all the conditions (AU, SO: *p* < 0.001; II: *p* < 0.001). Two-way analysis of variance (conditions and right/left positions as factors) indicated that main effects of the conditions and right/left positions as well as interactions between the two factors were not significant (Fig. 3, right).

### α suppression when observing the videos (the occipital region)

The results of a one-sample *t* test in the occipital region indicated significant α (10–12 Hz) suppression under all the conditions (AU, SO: *p* < 0.001; II: *p* < 0.001). Moreover, the results of one-way analysis of variance (the condition as a factor) did not indicate significant main effects of the condition. The results of a one-sample *t* test in the occipital region indicated significant α (8–10 Hz) suppression under all the conditions (AU: *p* < 0.001; II: *p* = 0.001; SO: *p* = 0.003). Moreover, the results of one-way analysis of variance (the condition as a factor) did not indicate significant main effects of the condition.

## Discussion

This study examined what type of action observation would most facilitate mirror system activities using brain waves. Comparison of the suppression amount of mu rhythms (8–10 Hz/10–12 Hz) when observing videos among three conditions, i.e., AU, II, and SO conditions, indicated that the suppression amount of mu (10–12 Hz) was largest in the central region under AU condition, compared to SO condition. Moreover, the suppression amount under AU condition was larger than that under II condition. AU condition includes the perception of social information, i.e., the conjecture of another person’s intention. Perry et al. reported mu suppression more increased when perceiving social context, compared to when judging the gender of the observation target [20]. It might be possible that mirror system activities were facilitated by a higher level request, i.e., perceiving social context, compared to SO or II condition.

In this study, participants observed the videos freely, without any limitation in the number of intentions of the action conceived under AU condition. The direct matching hypothesis, which explains the mechanism of action understanding, suggests that neurons in the ventral premotor cortex encode the goal of an action, and the goal is represented by the motor system of an observer when observing another person’s action, and thus the action is understood [24]. Under AU condition, more actions are represented in the brain, compared to II condition (imitating an action) and SO condition (simple observation), and mu suppression is supposed to be facilitated.

No significant differences were indicated between II and SO conditions. Some previous studies reported the activation level increased when observing with intending imitation, compared to simple observation [25, 26]. In these studies, imitation was performed during or after observing an action. In the present study, on the other hand, imitation was not performed just after observation, and only an instruction to observe with intending imitation was given. Since there were no differences among the conditions in the engagement in and attention to the task, participants are supposed to have watched the videos sufficiently following the instruction. However, the effect of the II condition might have been decreased because imitation was not performed.

Differences among the conditions were observed in the high-frequency band (10–12 Hz), whereas no differences were shown in the low-frequency band (8–10 Hz). The stimuli given in this study were actions of the right hand using a sponge by the fingers. High-frequency components of mu waves are considered to reflect finer movements, compared to low-frequency components [14]. Therefore, differences between the conditions might have more clearly reflected in 10–12 Hz. Moreover, there are some reports that social cognitive abilities reflected in mu waves are observed in the high-frequency band. For example, Naeem et al. indicated specific patterns in 10–12 Hz when intentional social coordination was made [16]. Furthermore, Hudac et al. reported that mu suppression patterns shown by autism spectrum disorder patients when observing an action were different from healthy controls, especially in 10–12 Hz [27]. Since AU condition requires social cognition, differences among conditions are considered to have been observed in 10–12 Hz.

On the other hand, significant mu suppression occurred also in 8–10 Hz during action observation. It might be possible that mu suppression by mirror system activation occurred also in the low-frequency band. Some studies suggested that mirror system activities are more reflected in the low-frequency band [28]. Further examination is required.

In the occipital region, no suppression was indicated in either high- or low-frequency band. Mu suppression when observing an action might be affected by α wave suppression in the occipital region, which fluctuates depending on the input of visual stimuli or attention [22]. The present study did not indicate the differences among conditions in the occipital region, which might increase the possibility that the results in the central region might be produced by mirror system activities.

## Conclusions

It was indicated that mu suppression when observing an action aiming to understand the intention of the action was larger, compared to simple observation as well as when observing an action aiming to imitate the action. It was suggested that mirror system activities are most activated when observing an action aiming to understand the intention of the action. Moreover, this tendency was shown only in 10–12 Hz and not shown in 8–10 Hz. The results above suggest that differences in the mirror system activity levels depending on the inner state might be shown in mu high-frequency band. In the future mirror system studies, differences depending on the frequency band should be examined in detail.

## Abbreviations

AU: Action understanding
EEG: Electroencephalogram
ERSP: Event-related spectral perturbation
II: Imaginarily imitation
SO: Simple observation
VAS: Visual analog scale
